# Analysis of selected crime data in Nigeria

**DOI:** 10.1016/j.dib.2018.05.143

**Published:** 2018-06-02

**Authors:** Pelumi E. Oguntunde, Oluwadare O. Ojo, Hilary I. Okagbue, Omoleye A. Oguntunde

**Affiliations:** a Department of Mathematics, Covenant University, Ota, Nigeria; b Department of Statistics, Federal University of Technology, Akure, Nigeria; c Department of Business Management, Covenant University, Ota, Nigeria

**Keywords:** Crime, Deviant behavior, Offences, Population, Poverty, Unemployment, Nigeria

## Abstract

Crime is an act that brings about offences and it is punishable under the law. Major crimes in Nigeria include rape, kidnapping, murder, burglary, fraud, terrorism, robbery, cyber-crimes, bribery and corruption, money laundering and so on. According to the statistics released by the Nigerian National Bureau of Statistics in 2016, Lagos, Abuja, Delta, Kano, Plateau, Ondo, Oyo, Bauchi, Adamawa and Gombe States made the top ten list of states with high number of crimes. Crime is an important topic and it is of interest to us because of the consequences and penalties it attracts (which ranges from fine to death). This data article contains the partial analysis (both descriptive and inferential) of crime data set obtained between 1999 and 2013. The aim of the study is to show the pattern and rate of crime in Nigeria based on the data collected and to show the relationships that exist among the various crime types. Analyzing this data set can provide insight on crime activities within Nigeria.

**Specifications Table**

**Table:** 

Subject area	Social Sciences
More specific subject area	Psychology, Criminology, Social Statistics
Type of data	Table and text file
How data was acquired	Secondary data
Data format	Raw, partial analyzed (Descriptive and Inferential)
Experimental factors	Data sets on some reported crime activities in Nigeria between 1999 and 2013 (21 years).
Experimental features	Observations on the number of cases of murder, arm robbery, assault, felonious wounding, man slaughter, bribery and corruption, burglary (including store breaking and house breaking).
Data source location	The data was obtained from Nigeria׳s Bureau of Statistics (NBS) database
Data accessibility	All the data are in this data article

**Value of the data**

• The data provide insight on crime activities and its study can help in crime reduction (protection of communities) and decision making.
• The partial analysis provided can be used to explain the relationships that exist between some of the crime activities.
• The data is useful in the following areas: criminology, sociology, psychology and statistics.
• The data can further be analyzed using other statistical methods like Principal Component Analysis (PCA), Panel data analysis and so on.

## 1 Data

The data in this article involves the reported cases of murder, arm robbery, assault, felonious wounding, man slaughter, bribery and corruption, burglary (including store breaking and house breaking) in Nigeria between the years 1999 and 2013. Ref. [1] has rated crime activities according to states in Nigeria and these criminal activities can be linked to poverty, unemployment, inflation, illiteracy, lack of education, greediness and over-population [2], [3], [4], [5], [6]. The study of crime is however very important because of its several implications on the society at large. Other studies on crime can be found in Refs. [7], [8], [9], [10], [11], [12], [13], [14], [15], [16], [17], [18], [19], [20] and the references therein.

The dataset used in this study was collected as a secondary data and it can be assessed as Supplementary data. The nature of the data is such that it can be analyzed using correlation analysis, principal component analysis, time series analysis and so on.

The summary of the data is as provided in Table 1.

**Table 1:** Summary statistics of the data set on crime activities.

**Crime types**	**Mean**	**Mode**	**Sum**	**Skewness**	**Kurtosis**
Murder	1814.48	1453^a^	38,104	1.056	0.565
Armed Robbery	2126.19	1064^a^	44,650	0.058	−1.317
Assault	43890.19	28,925^a^	921,694	−0.120	−1.271
Felonious Wounding	16667.05	9659^a^	350,008	1.257	4.317
Manslaughter	33.52	14^a^	704	0.935	0.074
Bribery and Corruption	208.00	10^a^	4368	0.878	−0.220
Burglary	22179.10	10,265^a^	465,761	3.520	14.231

a Denotes multiple mode. However, the smallest number is shown.

From Table 1, Assault has the highest number of cases (with a total of 921,694 reported cases) over the years considered, followed by Burglary.

**Fig. 1:**
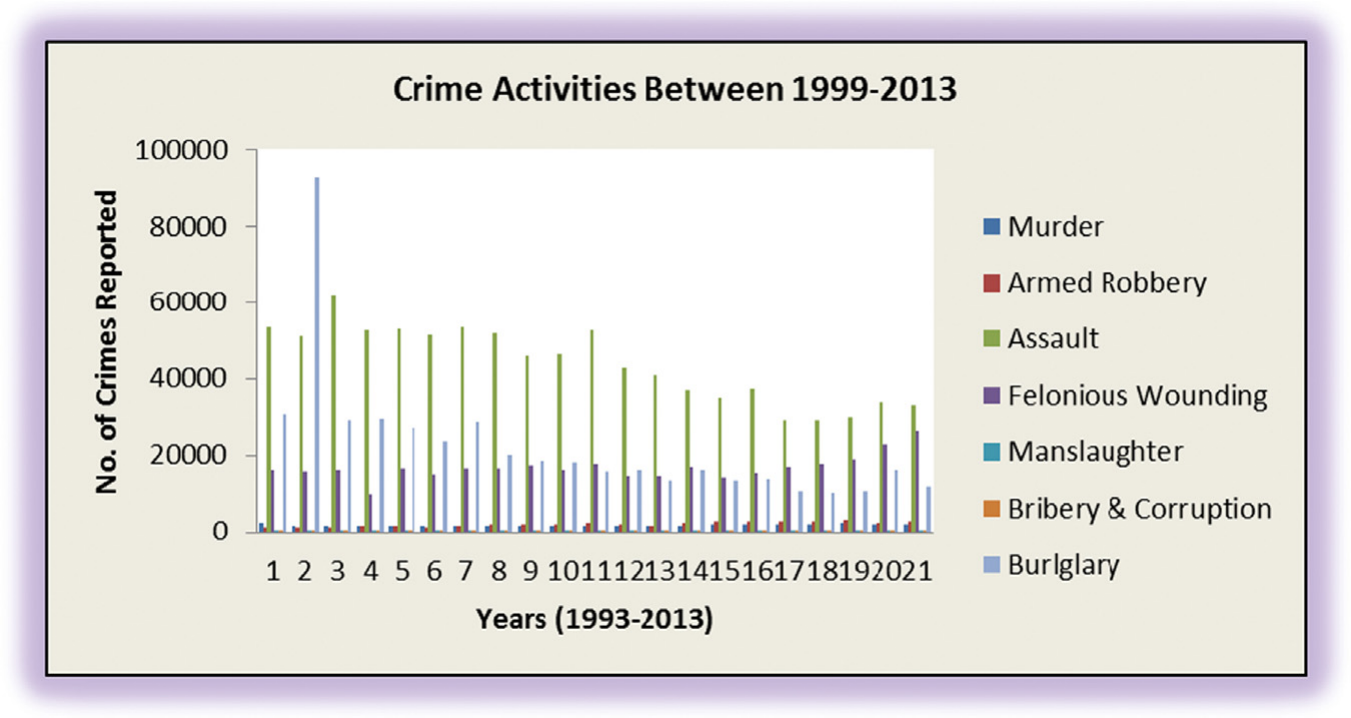
Graphical representation of the number of crime activities.

A graphical representation of the raw data is as shown in Fig. 1.

**Fig. 2:**
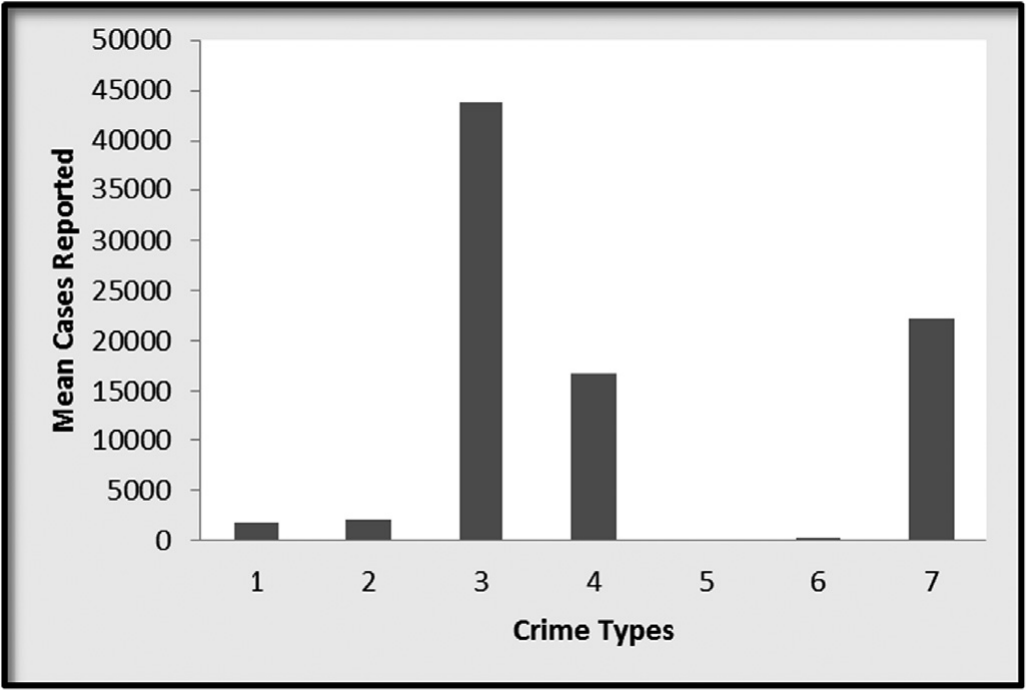
Graphical representation of the mean number of the different crime types.

Also, a graph representing the mean number of crimes reported for each of the crime types is as shown in Fig. 2.

From Fig. 2, Assault remains the most common type of crime reported based on its mean value followed by Burglary. The least among the crime types is Manslaughter. This also affirms the results in Table 1.

The pattern and trend of the crimes between 1999 and 2013 is made available in Fig. 3, Fig. 4, Fig. 5, Fig. 6, Fig. 7, Fig. 8, Fig. 9, Fig. 10.

**Fig. 3:**
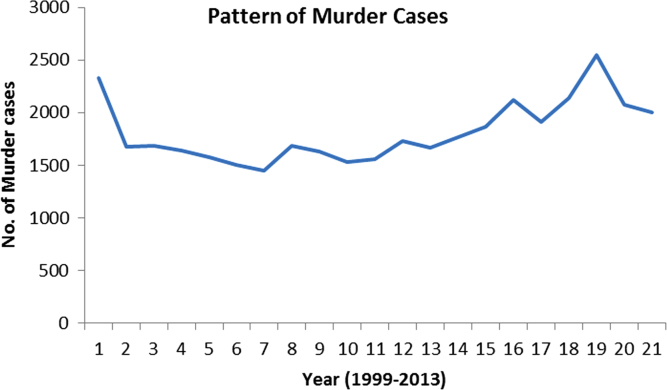
The pattern of murder cases for the 21 years.

**Fig. 4:**
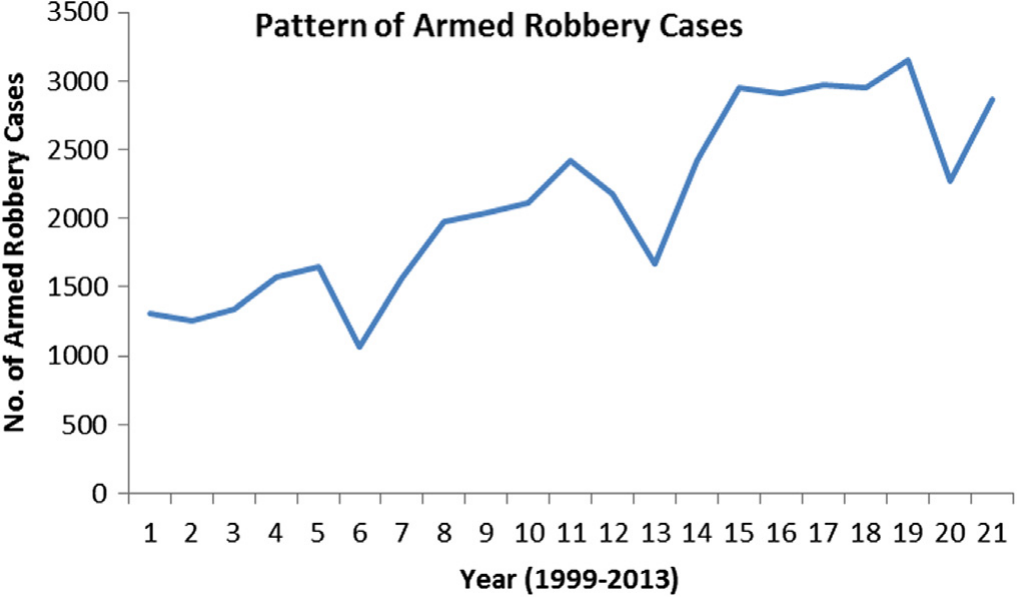
The pattern of armed robbery cases for the 21 years.

**Fig. 5:**
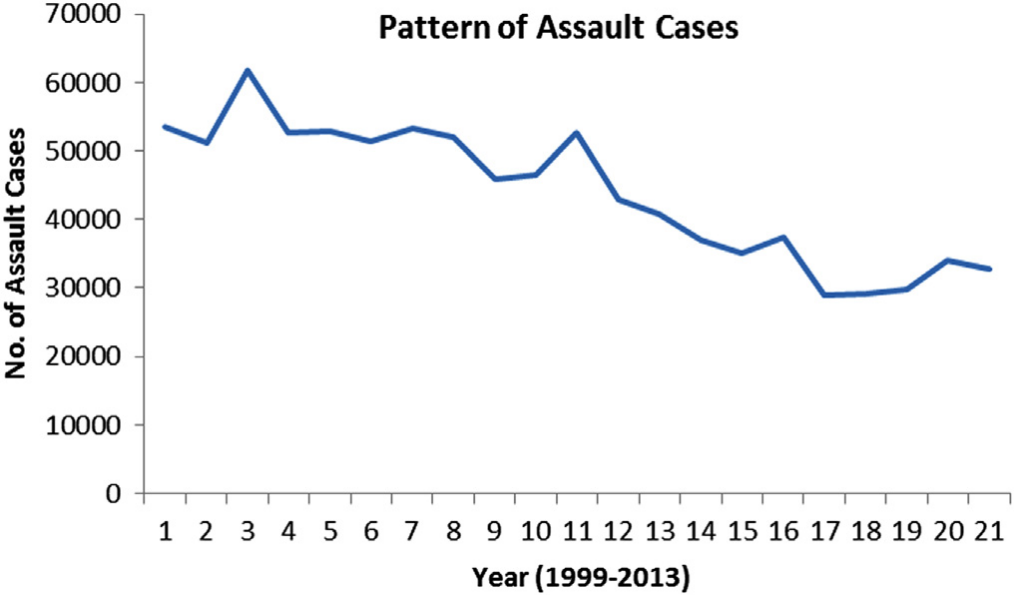
The pattern of assault cases for the 21 years.

**Fig. 6:**
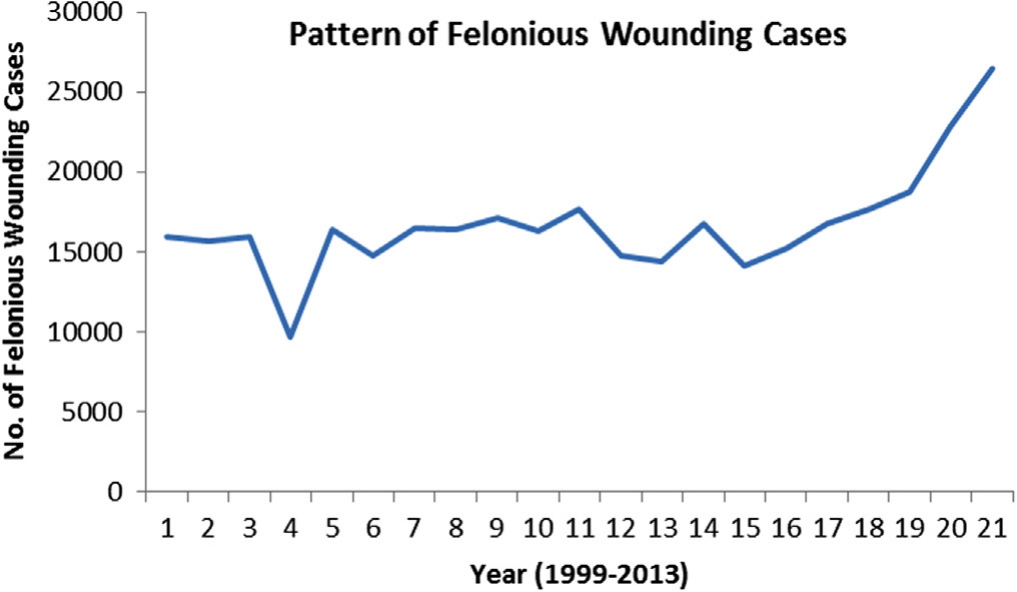
The pattern of felonious wounding cases for the 21 years.

**Fig. 7:**
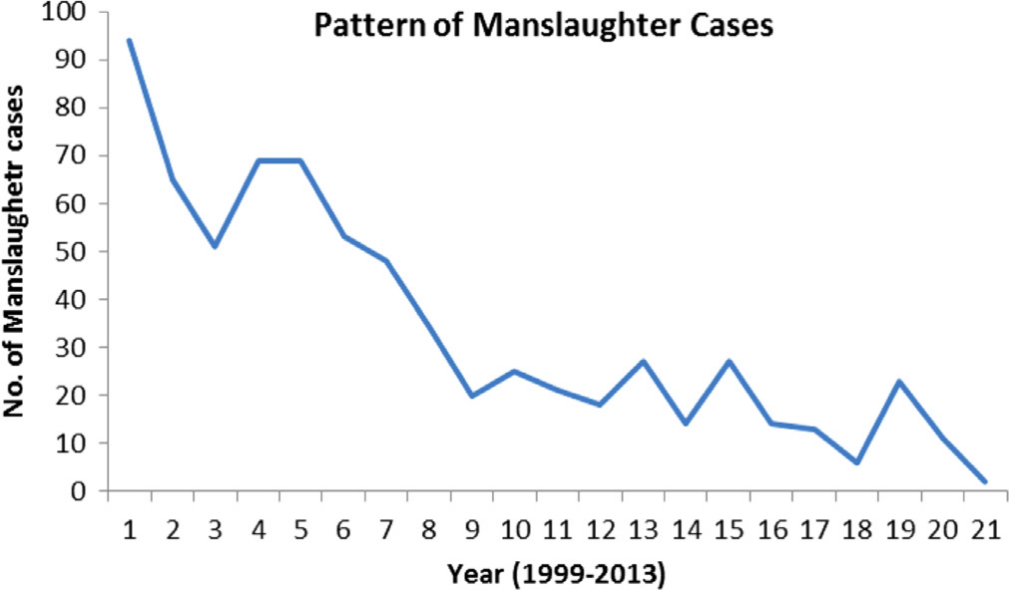
The pattern of man slaughter cases for the 21 years.

**Fig. 8:**
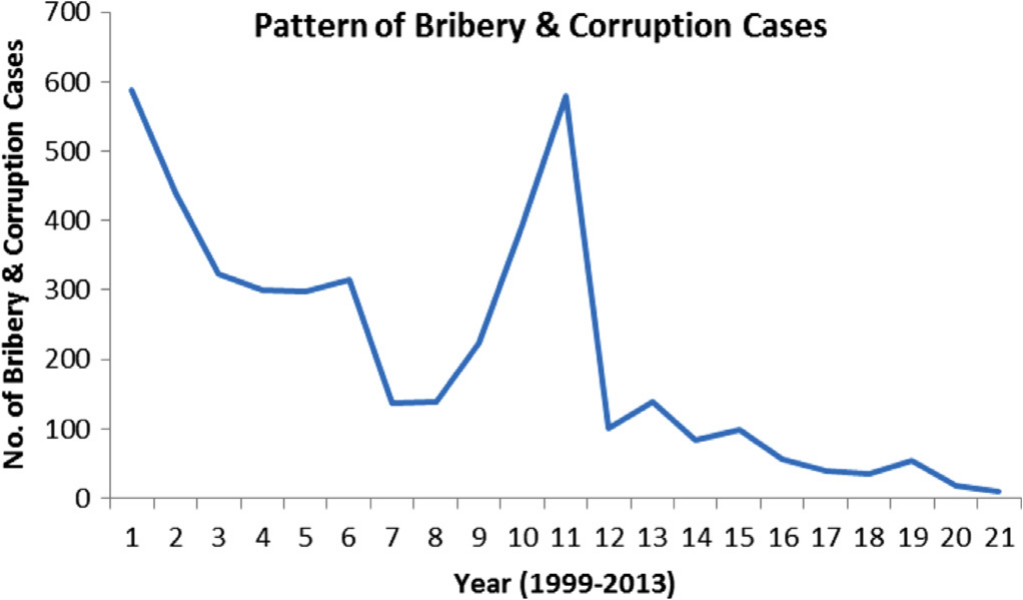
The pattern of bribery and corruption cases for the 21 years.

**Fig. 9:**
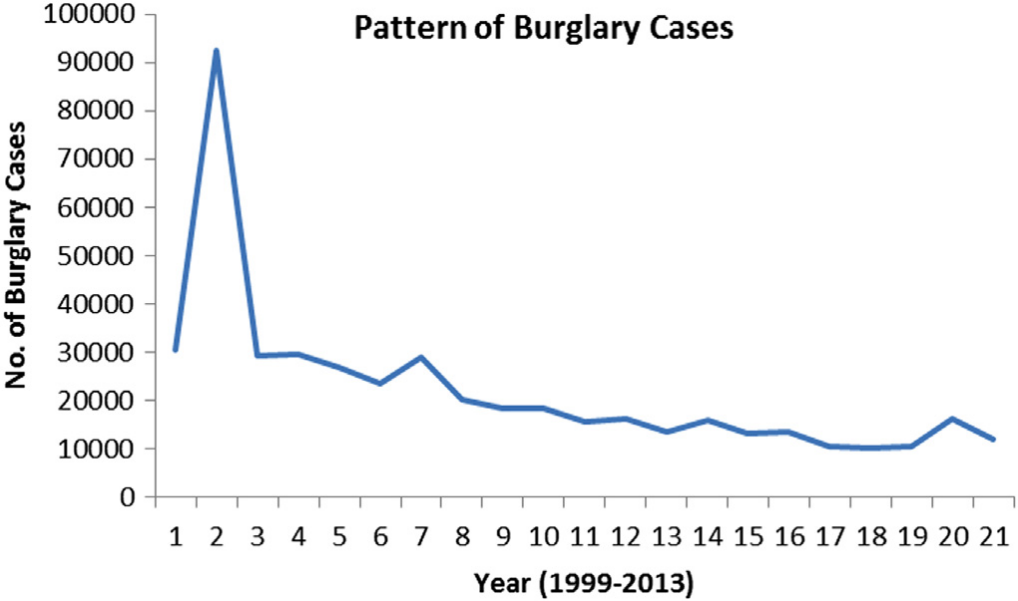
The pattern of burglary cases for the 21 years.

**Fig. 10:**
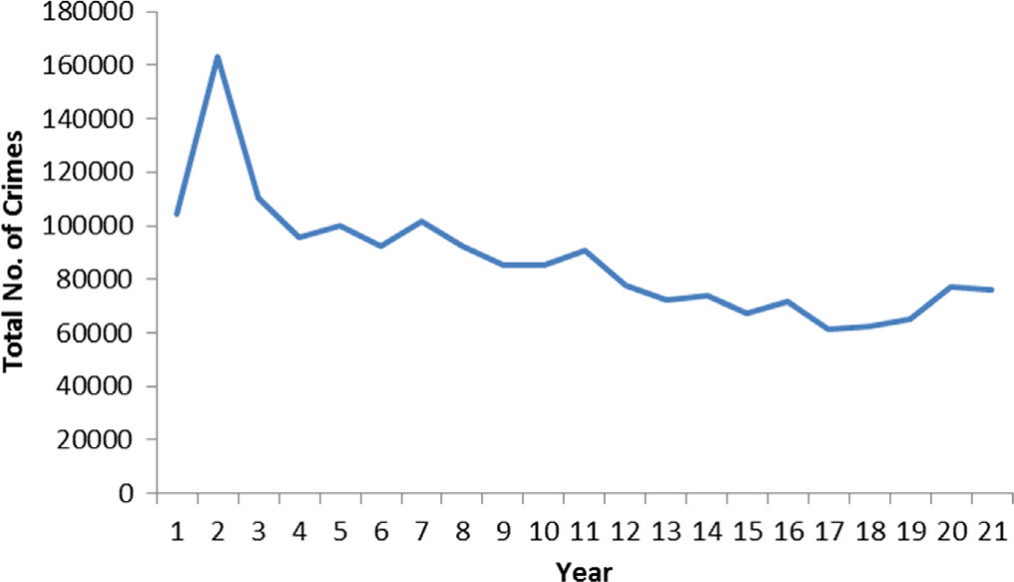
The pattern of crime (total) for the 21 years.

## 2 Experimental design, materials and methods

This article shows the strength of linear relationship that exists between crime activities using correlation analysis. It further tests whether the linear relationship is significantly different from zero or not. In particular, the hypothesis tested for the linear relationship between murder and armed robbery is:

H_0_: The linear relationship between murder and armed robbery is not significantly different from zero.

Versus

H_1_: The linear relationship between murder and armed robbery is significantly different from zero.

The null hypothesis is however rejected if the *p*-value is less or equal to the level of significance (0.05).

Other descriptive methods as contained in Refs. [21], [22], [23], [24] can also be used to explain the patterns and trend of the data set collected. The result for the correlation analysis is made available in Table 2.

**Table 2:** Results of the correlation analysis between the crime types (p-value in parenthesis).

	Murder	Armed Robbery	Assault	Felonious Wounding	Manslaughter	Bribery & Corruption	Burglary
Murder	1	**0.550**	**−0.605**	0.359	−0.184	−0.317	−0.251
		(0.010)	(0.004)	(0.110)	(0.425)	(0.162)	(0.272)
Armed Robbery		1	**−0.851**	0.402	**−0.792**	**−0.629**	**−0.597**
			(0.000)	(0.071)	(0.000)	(0.002)	(0.004)
Assault			1	−0.425	**0.746**	**0.756**	**0.492**
				(0.055)	(0.000)	(0.000)	(0.023)
Felonious Wounding				1	**−0.501**	−0.331	−0.221
					(0.021)	(0.142)	(0.336)
Manslaughter					1	**0.694**	**0.598**
						(0.000)	(0.004)
Bribery & Corruption						1	**0.497**
							(0.022)
Burglary							1

From Table 2, the results written in bold indicate a significant correlation among the pairs considered at 0.05 level of significance. We can however say that:

• The positive linear relationship between murder and armed robbery is significantly different from zero.
• The negative linear relationship between murder and armed assault is significantly different from zero.
• The negative linear relationship between armed robbery and assault is significantly different from zero
• There negative linear relationship between armed robbery and man slaughter is significantly different from zero.
• The negative linear relationship between armed robbery and bribery and corruption is significantly different from zero.
• There is a significant positive linear relationship between assault and man slaughter.
• There is a significant positive linear relationship between assault and bribery and corruption.
• There is a significant positive linear relationship between assault and burglary.
• There is a significant negative linear relationship between felonious wounding and man slaughter.
• There is a significant positive linear relationship between man slaughter and bribery and corruption.
• There is a significant positive linear relationship between man slaughter and burglary.
