# Supplementary material for: Analysis of selected crime data in Nigeria
Source: Data Brief. 2018 Jun 2;19:1242–9. doi: 10.1016/j.dib.2018.05.143 (PMC6140829; doi:10.1016/j.dib.2018.05.143)
Supplement: Supplementary file 1 — Supplementary material. [file mmc1.pdf]

COVENANT UNIVERSITY
COLLEGE OF SCIENCE AND TECHNOLOGY
DEPARTMENT OF MATHEMATICS

CANAANLAND, KM 10, IDIROKO ROAD
P.M.B 1023, OTA, OGUN STATE, NIGERIA
www.covenantuniversity.edu.ng, mat.covenantuniversity.edu.ng

EXTERNAL MEMO

To: Editor, Data in Brief
From: Corresponding Author
Date: 26th May, 2018
Subject: **Conflict of Interest**

I thereby declare the absence of any conflict of interest among the authors.

The authors have read the final draft and unanimously agreed that the paper be sent for review.

The source of funding and data have been acknowledged.

Dr. Pelumi E. Oguntunde

pelumi.oguntunde@covenantuniversity.edu.ng

Department of Mathematics,

Covenant University, Ota, Nigeria
